# Longitudinal association between the timing of adiposity peak and rebound and overweight at seven years of age

**DOI:** 10.1186/s12887-022-03190-9

**Published:** 2022-04-19

**Authors:** Dan Lin, Di-di Chen, Jun Huang, Yun Li, Xiao-sa Wen, Hui-jing Shi

**Affiliations:** 1 Department of Maternal, Child and Adolescent Health, School of Public Health, Fudan University, Shanghai, China; 2 Minhang District Centre of Disease Control and Prevention, Shanghai, China; 3 Minhang Branch, School of Public Health, Fudan University, Shanghai, China; 4 Minhang Maternal and Child Health Centre, Shanghai, China

**Keywords:** Body mass index (BMI), Obesity, Overweight, Children, Adiposity peak, Adiposity rebound

## Abstract

**Background:**

The timing of adiposity peak (AP) or adiposity rebound (AR) is a determinant of overweight or obesity in adolescence and adulthood. However, limited studies have reported the association in young school-age children. We aimed to evaluate this association and explore the role of health behaviours in it.

**Methods:**

Routinely collected, sequential, anthropometric data from the 1st to 80th months of age were used to estimate AP and AR timings in 2330 children born in Shanghai between 2010 and 2013. Multivariate regression analyses were applied to identify the associations between the AP or AR timings and the risk of developing overweight or obesity in first-grade school children. The roles of health behaviours, including dietary patterns, physical activity level, sleep and snacking habits, and screen time, were also evaluated.

**Results:**

Children with a late AP or an early AR were at higher risk of overweight but not obesity or central obesity in their first grade. A high physical activity level was associated with a lower risk of having overweight in children with a late AP, and limited screen time was associated with a decreased risk of having overweight or obesity in children with an early AR. The absence of a late-night snacking habit in children with a non-early AR indicated a decreased risk of having overweight. However, this association was not observed among children with an early AR.

**Conclusion:**

The timings of AP and AR are tied to overweight in middle childhood. Prevention strategies are suggested to move forward to control late AP and early AR.

**Supplementary Information:**

The online version contains supplementary material available at 10.1186/s12887-022-03190-9.

## Introduction

The worldwide prevalence of overweight or obesity among children has increased dramatically over the last few decades, and childhood obesity has become a pandemic [1–6]. Although the prevalence has appeared to plateau in developed countries [7–11], it continues to increase in developing nations [1, 12]. Due to the rapid transitions in dietary patterns and lifestyles in China, a growing number of children affected by overweight or obesity have been identified in recent years [13]. Nationally representative data indicated that the prevalence of overweight or obesity in school-age children was approximately 30% in 2016 [14]. Childhood overweight or obesity leads to long-term serious health consequences for individuals, and strong evidence indicates associations between childhood obesity and non-communicable diseases, cardiometabolic risk factors, and mental disorders later in life [15–20]. Additionally, the continuation of overweight or obesity status from childhood to adulthood enhances the risk of morbidity and mortality [21, 22], and childhood overweight and obesity lead to a national burden on the medical system for lifetime health care costs [23]. Given the long-term adverse health outcomes for these individuals and the disease burdens on the nation, it is urgent to clarify the evidence for obesity-associated putative factors in the early stages of life and highlight the importance of primary prevention for halting the rapidly rising trend.

Overweight or obesity is defined as abnormal body fat accrual and is most commonly assessed using body mass index (BMI) [24]. Reviewing the BMI trajectory in individuals and tracking the dynamics of BMI change over time from an early age is considered to be effective in predicting excess weight gain later in life [25–29]. After birth, the BMI-for-age trajectory rapidly increases to a peak at six to 12 months of age and then declines to the nadir between the ages of four and six years. After this decline, the trajectory gradually rises again through adolescence and most of adulthood [30, 31]. The highest point of the BMI trajectory during infancy is termed the “adiposity peak (AP)”, and the nadir is referred to as the “adiposity rebound (AR)” [26]. Previous studies have shown that the timing of the AP and AR are indicators predicting later fatness [32–38]. A late AP or an early AR has been suggested to be associated with an increased risk of overweight or obesity, glucose intolerance, and metabolic syndrome in adolescence and adulthood [38–48]. However, limited studies have directly reported the association between a late AP or an early AR and the BMI value or the risk of developing overweight/obesity in school-age children (six to 12 years) [49–54].

Experts argue that the major predisposing factors for childhood obesity are present at an early age and cluster with obesity-related behaviours, such as physical activity (PA) and eating habits [27, 29, 55–57]. These essential but modifiable behaviours could also independently affect the BMI outcomes of children [58–61]. Nevertheless, little attention has been given to the association between health behaviours in children with a late AP or an early AR and the risk of having overweight or obesity during the school-age years.

To fill these gaps, we conducted a retrospective study in children who were born and living in Shanghai, China, aiming to address two research goals: (1) To evaluate whether a late AP or an early AR is associated with a higher risk of developing overweight or obesity in young school-age children; (2) To explore whether positive health behaviour is tied to a reduced risk of developing overweight or obesity among children with a late AP or an early AR. This study was used to test the hypothesis that children with a late AP or an early AR will have a higher risk of overweight or obesity during their early school age. However, that risk will be reduced for those with positive health behaviours such as healthy dietary patterns, moderate to high PA levels, good sleeping and snacking habits, or limited screen time.

## Methods

### Study population

The potential study participants were children born in Minhang, Shanghai, between 1 September 2010 and 31 October 2013. In the 2019/2020 academic year, they entered the first grade of local primary schools (*N* = 14 450). We retrieved recorded data on the age, sex, height, and weight of these children from well-child visits or other consultations reported by clinics and hospitals in this area from the Health Commission-authorized database. Every anthropometric measurement taken for the children was recorded by the health care professionals. We only selected children who had at least three anthropometric measurements (weight and length/height at the same assessment and only one measurement per month) during their 1st and 80th months of age for growth trajectory fitting to identify their AP and AR timing [62]. Additionally, information concerning the parental ages at delivery, parental education levels, gestational weeks, delivery modes, birth weight and birth length was retrieved from their birth records. In 2019, the participants received their first school health checks by qualified professionals from community health centres in their first semester, where anthropometric parameters were measured. We have randomly selected 17 primary schools in Minhang and matched 3533 subjects according to the list of potential participants in that area. An electronic questionnaire about socio-demographic characteristics and lifestyle/behaviour was sent to the parents or caregivers for completion by the children’s headteachers during the week of the school health check. Oral informed consent was obtained from all children who attended the health check, and electronic informed consent was obtained from the parent or caregiver who filled out the questionnaire. Ethical approval was given by the Minhang District Centers for Disease Control and Prevention Ethics Committee, with the approval number EC-2019–011.

### Participant selection

**Fig. 1:**
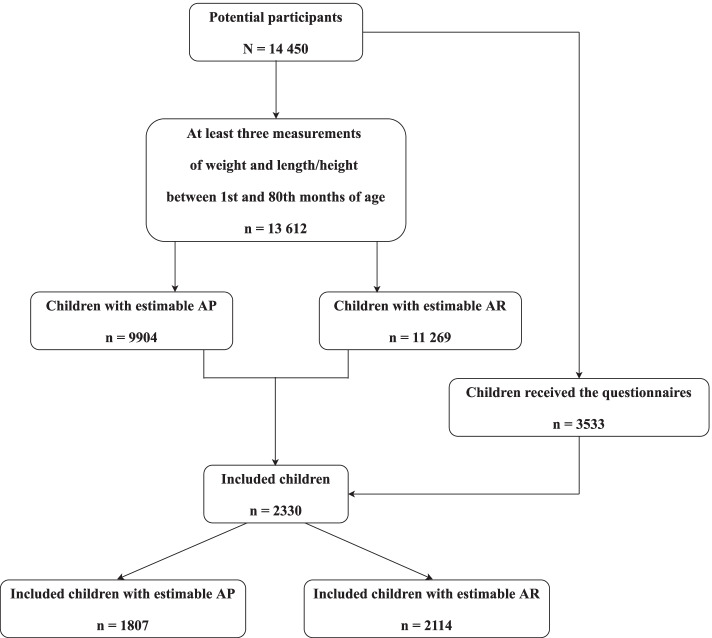
Study flow chart of participants selection

Detailed information on the selection criteria is depicted in Fig. 1. The growth data were available in 13 612 children who had at least three anthropometric measurements during their first and 80th months of age. Their growth trajectories were fitted [62], and the AP and AR could not be estimated for 3708 and 2343 children, respectively. In our study, 9904 children with estimable AP and 11 269 children with estimable AR were found. The children’s headteachers helped ensure that the parents or caregivers of the 3533 children all finished the electronic questionnaire and completed the online submission. We then matched the children with estimable AP/AR with those whose parents completed the electronic questionnaires. Finally, a total of 2330 eligible children were included.

### Timing of AP and AR

All data for multiple observations obtained in early life regarding children’s ages, weights, and lengths/heights were used to estimate BMI and its trajectories (at least three BMI assessments per child during their first and 80th months of age and only one assessment per month) [62]. The individual-specific BMI trajectory from the first to 80th months of age was fitted using nonlinear mixed-effect models with natural cubic spline functions for age to capture the nonlinear trend [63]. Estimates of age at the AP and AR were drawn from the BMI-for-age curve for each child. Based on these estimates, 1807 and 2114 children had their ages estimated for the AP and AR, respectively. In this study, the timings of AP and AR were set as binary variables. According to the literature, a child who had an AP occurrence later than nine months of age was defined as having a late AP, and a child who had an AR occurrence earlier than 60 months of age was defined as having an early AR [43, 45, 46, 64].

### Overweight or obesity in first-grade primary school children

The endpoint of our study was the status of overweight or obesity in children in first grade. The participants’ data on age, sex, height, weight, and waist circumference (WC) were collected from the school health check. All practices were strictly followed for the Management Measures for Physical Examination of Primary and Secondary School Students in China, and trained nurses who participated in in-service training performed all measurements following standardized techniques to ensure validity. Bodyweight and height were measured by calibrated mechanical weight scales and stadiometers, respectively, which passed the inspection of the Shanghai Compulsory Verification Center for Measuring Instruments. Weight was measured approximately to the nearest 100 g, and height was measured approximately to the nearest 0.5 cm. BMI was calculated as body weight in kilograms divided by length or height in meters squared. WC was measured to the nearest 0.1 cm by a nonelastic flexible tape with the child in a standing position. The tape was applied horizontally midway between the lowest rib margin and the iliac crest. We defined overweight and obesity based on the age- and sex-specific BMI cutoffs using the International Obesity Task Force (IOTF) definition [65]. The waist-to-height ratio (WHTR) was calculated by dividing WC by height, and 0.50 was applied as a cutoff for defining central obesity [66].

### Covariates

The covariates in this study included sex, race, age at the school health check, birth weight, preterm birth (yes or no), delivery mode (natural or cesarean), breastfeeding duration (longer than four months or not), parental ages at childbirth, parental education levels (university-educated or not), parents’ self-reported prepregnancy BMIs (normal weight, overweight or obesity), number of people in the household, number of siblings (0 or ≥ 1), parents’ perceived household income level (poor, about the same, rich), dietary patterns (processed foods, traditional foods, or light meals), current PA level (low, moderate or high) [67, 68], sleep disturbances (yes or no), lack of sleep (yes or no), screen time (< two hours per day or longer), and late-night snacking habits (snacking within one hour before going to bed, yes or no). The data about birth and feedings were extracted from the birth and postpartum-visit records. Data about socio-demographic characteristics and health or lifestyle behaviours were collected by electronic questionnaires.

The dietary patterns were estimated from a food frequency questionnaire (FFQ) answered by the parents or caregivers. We adopted an FFQ that was previously used for school-age children in a neighbouring province, and the Cronbach's alpha coefficient was 0.86 [69]. Habitual dietary patterns, which were derived from 28 standardized food items by principal component analysis (PCA), were assessed by the parent-reported FFQ to measure the children’s dietary habits during the last month before the school health check [70, 71]. The times of consumption for each food or food group per week were reported. There were six frequency options, ranging from “never” to “three or more times per day”. The FFQ only focused on the frequency of food intake, and portion size or energy was not included. We then used the habitual dietary patterns to categorize the participants. Exploratory PCA was performed on the correlation matrix of the foods (Supplemental Tables 1 and 2). To determine the number of components for retention, we considered a combination of the interpretability of the factors and the proportion of total explained variance [72]. Varimax (orthogonal) rotation was applied to the factor loadings matrix to facilitate their interpretability [70]. Foods with rotated factor loadings greater than or equal to 0.30 in a given factor were used to label the factor [73]. The number of components best representing the data was chosen based on the interpretability of the patterns. A component score was created for each child for each of the identified components. The scores were a weighted linear combination of the food items, such that the weights determined how essential each food item was for that score. Each score had a mean of zero, and a higher score indicated closer adherence to that dietary pattern [74]. Each subject received a factor score for each extracted component, with a higher score indicating relatively higher adherence to that dietary pattern.

We used the last seven days for the short, self-administered version of the International Physical Activity Questionnaire (IPAQ) to measure health-related PA levels [67, 68]. We calculated the children’s overall scores by the scoring protocol and divided the population into three categories (low, moderate, or high PA levels). However, in our questionnaire, the answer was given by the parents or caregivers. We also used the Children's Sleep Habits Questionnaire (CSHQ) – Abbreviated form to examine major medical and behavioural sleep disorders for these school-age children [75–77]. Scores over 41 are considered to indicate that a child has sleep disturbances. The Cronbach's alpha coefficient was 0.86 in a prior Chinese study [76]. Sleep duration (i.e., the length of sleep time per day) was also collected, and less than ten hours per day was used as the cutoff for lack of sleep [78]. The positive health behaviours listed in this study included the habitual dietary patterns of traditional foods or light meals (compared to the dietary pattern of processed foods), moderate or high levels of PA (compared to low levels), high quality of sleep, sufficient sleep (≥ ten hours per day), the absence of a late-night snacking habit, and limited screen time (< two hours per day).

### Statistical analysis

We tested the distribution of our continuous variables and found that it was nonnormal. Hence, data are presented as medians (interquartile ranges, IQRs) for continuous variables and frequencies (percentages) for categorical variables. Differences of characteristics among children with different AP or AR timing were detected by the Kruskal–Wallis H test for continuous variables and the chi-square test for dichotomous variables.

We used logistic regression models or generalized linear models (GLMs) to assess the association between a late AP or an early AR and the odds or risk of having overweight or obesity reported in the first grade among these children (the model selection depended on the detection rate for overweight or obesity in each outcome category; if the detection rate was > 15%, we applied GLMs, or we chose a logistic regression model) [79]. Odds ratios (ORs) or risk ratios (RRs) with their 95% confidence intervals (95% CIs) were estimated. Furthermore, based on the obtained associations, we attempted to explore whether positive health behaviours could independently change the risk or likelihood of developing overweight or obesity during middle childhood in children with a late AP or an early AR. The full models were applied, and the adjusted covariates included age at the health check, sex, birth weight, gestational age (preterm birth), delivery mode, breastfeeding duration, parental ages at birth, parental prepregnancy BMI statuses, parental education levels, perceived household income level, number of family members, number of siblings, dietary patterns, PA level, sleep disturbances, sleep duration, screen time, late-night snacking habits, and BMI magnitudes at the AP and AR. Sensitivity analyses were conducted, and the number of health outcomes was enlarged in children affected by overweight or obesity in the first grade. A *P* value < 0.05 (two-sided) was considered to indicate statistical significance. All statistical analyses were conducted using Stata 15.0 statistical software (StataCorp LP, College Station, TX, USA).

## Results

### Characteristics of the participants

For a total of 2330 eligible children, a median of 12 (interquartile range = 7) times of BMI measurements per child was available (the minimum was three measurements, and the maximum was 26 measurements per child). All parents or caregivers of these children completed the questionnaires. The characteristics of the participants with different timings of AP and AR are outlined in Table 1. Children with a late AP were more likely to have lower birth weights (3305 g vs 3418 g) and to be breastfed longer than four months (29.88% vs 20.21%). In the first grade in primary school, these children had larger body weights (23.60 kg vs 23.35 kg), BMI values (15.72 vs 15.61 kg/m^2^), and WCs (54 cm vs 53 cm). However, these children were less likely to have obesity or central obesity (4.81% vs 12.69%, 7.60% vs 14.18%, respectively).

**Table 1:** Characteristics of the children with a late AP or an early AR

Characteristics	Children with a late AP				Children with an early AR			
	**Yes**	**No**	**Total**	***P* value**	**Yes**	**No**	**Total**	***P* value**
	**(*N* = 1539)**	**(*N* = 268)**	**(*N* = 1807)**		**(*N* = 1814)**	**(*N* = 300)**	**(*N* = 2114)**	
Age — years	6.75 (6.42–6.92)	6.75 (6.50–7.00)	6.75 (6.42–6.92)	0.37	6.75 (6.42–6.92)	6.92 (6.67–7.00)	6.75 (6.50–7.00)	** < 0.001**
Male sex — no. (%)	779 (50.62)	146 (54.48)	925 (51.19)	0.24	974 (53.69)	142 (47.33)	1116 (52.79)	**0.04**
Race — no. (%)				0.91				0.10
Han	1497 (97.27)	261 (97.39)	1758 (97.29)		1771 (97.63)	288 (96.00)	2059 (97.40)	
Other	42 (2.73)	7 (2.61)	49 (2.71)		43 (2.37)	12 (4.00)	55 (2.60)	
BMI measurement during 1^st^ and 80^th^ months of age — times	12 (6–14)	13 (11–14)	13 (7–14)	** < 0.001**	13 (9–14)	7 (4–14)	13 (8–14)	** < 0.001**
Weight — kg	23.60 (21.50–26.80)	23.35 (21.30–27.63)	23.60 (21.40–26.90)	**0.04**	24.00 (21.70–27.10)	22.80 (20.98–25.63)	23.80 (21.50–26.90)	** < 0.001**
Height — cm	123 (119–127)	123 (119–126.58)	123 (119–127)	0.70	123 (119–126)	123 (120–127)	123 (119–127)	0.28
BMI — kg/m^2^	15.72(14.74–17.13)	15.61(14.64–17.24)	15.71(14.72–17.14)	**0.03**	15.90(14.80–17.40)	15.12(14.24–16.19)	15.76(14.74–17.15)	** < 0.001**
WC — cm	54 (51–57)	53 (51–58)	54 (51–57)	**0.02**	54 (51–58)	53 (50–56)	54 (51–57)	** < 0.001**
WHTR	0.44 (0.42–0.46)	0.44 (0.42–0.47)	0.44 (0.42–0.46)	**0.02**	0.44 (0.42–0.47)	0.43 (0.41–0.45)	0.44 (0.42–0.47)	** < 0.001**
Dietary pattern — no. (%)				0.40				0.63
Processed	947 (61.53)	175 (65.30)	1122 (62.09)		1109 (61.14)	187 (62.33)	1296 (61.31)	
Traditional	507 (32.94)	77 (28.73)	584 (32.32)		588 (32.41)	98 (32.67)	686 (32.45)	
Light meal	85 (5.52)	16 (5.97)	101 (5.59)		117 (6.45)	15 (5.00)	132 (6.24)	
Physical activity level — no. (%)				0.40				0.99
Low	1006 (65.37)	182 (67.91)	1188 (65.74)		1188 (65.49)	196 (65.33)	1384 (65.47)	
Moderate	361 (23.46)	53 (19.78)	414 (22.91)		425 (23.43)	70 (23.33)	495 (23.42)	
High	172 (11.18)	33 (12.31)	205 (11.34)		201 (11.08)	34 (11.33)	235 (11.12)	
Sleep disturbances — no. (%)	1259 (81.81)	212 (79.10)	1471 (81.41)	0.29	1475 (81.31)	250 (83.33)	1725 (81.60)	0.40
Lack of sleep — no. (%)	1186 (77.67)	204 (77.86)	1390 (77.70)	0.94	1402 (78.19)	231 (77.52)	1633 (78.10)	0.79
Screen time (≥ 2 h per day) — no. (%)	218 (14.17)	29 (10.82)	247 (13.67)	0.14	258 (14.22)	32 (10.67)	290 (13.72)	0.10
Late night snacking — no. (%)	558 (36.26)	103 (38.43)	661 (36.58)	0.50	667 (36.77)	97 (32.33)	764 (36.14)	0.14
People in their household	3 (2–4)	3 (2–4)	3 (2–4)	0.36	3 (2–4)	3 (2–4)	3 (2–4)	0.46
Number of siblings (≥ 1) — no. (%)	644 (41.85)	117 (43.66)	761 (42.11)	0.59	746 (41.12)	138 (46.00)	884 (41.82)	0.11
Maternal pre-pregnancy BMI status (overweight or obesity) — no. (%)	174 (11.31)	31 (11.57)	205 (11.34)	0.90	199 (10.97)	25 (8.33)	224 (10.60)	0.17
Paternal pre-pregnancy BMI status (overweight or obesity) — no. (%)	171 (11.11)	35 (13.06)	206 (11.40)	0.35	207 (11.41)	21 (7.00)	228 (10.79)	**0.03**
Perceived household income level — no. (%)				**0.01**				0.60
Poor	76 (4.94)	18 (6.72)	94 (5.20)		89 (4.91)	16 (5.33)	105 (4.97)	
About the same	1113 (72.32)	210 (78.36)	1323 (73.22)		1350 (74.42)	215 (71.67)	1565 (74.03)	
Richer	350 (22.74)	40 (14.93)	390 (21.58)		375 (20.67)	69 (23.00)	444 (21.00)	
Birth weight — g	3305 (3050–3590)	3418 (3150–3700)	3330 (3070–3600)	** < 0.001**	3360 (3100–3620)	3320 (3100–3600)	3350 (3100–3614)	0.98
Birth length — cm	50 (50–50)	50 (50–50)	50 (50–50)	0.11	50 (50–50)	50 (50–50)	50 (50–50)	0.15
Maternal age — years	29 (27–32)	29 (27–31)	29 (27–32)	0.31	29 (27–31)	29 (27–31)	29 (27–31)	0.44
Paternal age — years	31 (28–34)	30 (28–34)	31.00 (28–34)	0.80	31 (28–34)	31 (28–34)	31 (28–34)	0.73
Mother university-educated — no. (%)	1120 (81.75)	189 (81.47)	1309 (81.71)	0.92	1286 (80.43)	218 (83.21)	1504 (80.82)	0.29
Father university-educated — no. (%)	1152 (83.72)	187 (82.38)	1339 (83.53)	0.61	1328 (82.95)	223 (84.47)	1551 (83.16)	0.54
Preterm birth — no. (%)	62 (4.03)	9 (3.36)	71 (3.93)	0.60	72 (3.97)	6 (2.01)	78 (3.69)	0.10
Cesarean delivery — no. (%)	730 (47.46)	133 (49.81)	863 (47.81)	0.48	879 (48.51)	125 (41.67)	1004 (47.54)	**0.03**
Breastfeeding duration > 4 months — no. (%)	242 (29.88)	38 (20.21)	280 (28.06)	** < 0.01**	312 (28.57)	47 (43.93)	359 (29.94)	** < 0.001**
**Outcomes — no. (%)**								
Overweight or obesity	294 (19.10)	61 (22.76)	355 (19.65)	0.16	408 (22.49)	18 (6.00)	426 (20.15)	** < 0.001**
Overweight	220 (14.29)	27 (10.07)	247 (13.67)	0.14	297 (16.37)	13 (4.33)	310 (14.66)	** < 0.001**
Obesity	74 (4.81)	34 (12.69)	108 (5.98)	**0.01**	111 (6.12)	5 (1.67)	116 (5.49)	** < 0.01**
Central obesity	117 (7.60)	38 (14.18)	155 (8.58)	** < 0.001**	163 (9.00)	13 (4.35)	176 (8.34)	** < 0.01**

The total includes all children with estimated AP or AR timing. BMI, body mass index. Values are median (IQR) or number (%). *P*-value in bold font indicates a significant difference

Children with an early AR were more likely to be male (53.69% vs 47.33%) and have a father affected by overweight or obesity (11.41% vs 7.00%). These children were less likely to be born naturally (41.67% vs 48.51%) and breastfed longer than four months (28.57% vs 43.93%). In the first grade in primary school, they tended to have larger body weights (24.00 kg vs. 22.80 kg), BMI values (15.90 kg/m^2^ vs. 15.12 kg/m^2^), WCs (54 cm vs. 53 cm), and WHTRs (0.44 vs. 0.43). These children also had a higher likelihood of being affected by overweight (16.37% vs 4.33%), obesity (6.12% vs 1.67%), or central obesity (9.00% vs 4.35%).

### The association between timing of AP or AR and the risk of developing overweight or obesity for children in the first grade of primary school

Tables 2 and 3 show the odds or risks of having overweight or obesity in the first grade of primary school for children with a late AP or an early AR. In the full model adjusted for birth weight, breastfeeding duration, perceived household income level, age at AR, BMI at AP, and BMI at AR, children with a late AP were at higher risk of having overweight (*RR* = 2.30, 95% CI: 1.29–4.08, *P* < 0.01) but not obesity or central obesity in the first grade (Table 2). In the full model adjusted for age, sex, paternal prepregnancy BMI status, delivery mode, breastfeeding duration, age at AP, BMI at AP, and BMI at AR, children with an early AR were at higher risk of having overweight (*RR* = 5.11, 95% CI: 1.26–20.70, *P* < 0.02) but not obesity or central obesity in the first grade (Table 3).

**Table 2:** Association between timing of AP and overweight or obesity in children in the first grade of primary school

		**Overweight or obesity**		**Overweight**		**Obesity**		**Central obesity**	
		**RR (95% CI)**	***P*-value**	**RR (95% CI)**	***P*-value**	**OR (95% CI)**	***P*-value**	**OR (95% CI)**	***P*-value**
**Crude model**	Children with a non-late AP	reference							
	Children with a late AP	0.84 (0.66, 1.07)	0.16	1.42 (0.97, 2.07)	0.07	0.35 (0.23, 0.53)	** < 0.001**	0.50 (0.34, 0.74)	** < 0.001**
**Full model**	Children with a non-late AP	reference							
	Children with a late AP	1.20 (0.86, 1.68)	0.28	2.30 (1.29, 4.08)	** < 0.01**	0.48 (0.18, 1.27)	0.14	0.88 (0.41, 1.89)	0.75

*OR* odds ratio, *RR* risk ratio, *CI* confidence interval. The crude model was unadjusted. The full model was adjusted for birth weight, breastfeeding duration, perceived household income level, age at AR, BMI at AP, and BMI at AR. *P*-value in bold font indicates a significant difference

**Table 3:** Association between timing of AR and overweight or obesity in children in the first grade of primary school

		**Overweight or obesity**		**Overweight**		**Obesity**		**Central obesity**	
		**RR (95% CI)**	***P*-value**	**RR (95% CI)**	***P*-value**	**OR (95% CI)**	***P*-value**	**OR (95% CI)**	***P*-value**
**Crude model**	Children with a non-early AR	reference							
	Children with an early AR	3.75 (2.38, 5.91)	** < 0.001**	3.77 (2.20, 6.50)	** < 0.001**	3.85 (1.56, 9.50)	** < 0.01**	2.17 (1.22, 3.88)	**0.01**
**Full model**	Children with a non-early AR	reference							
	Children with an early AR	4.39 (1.45, 13.26)	** < 0.01**	5.11 (1.26, 20.70)	**0.02**	2.72 (0.32, 22.99)	0.36	1.34 (0.37, 4.77)	0.66

*OR* odds ratio, *RR* risk ratio, *CI* confidence interval. The crude model was unadjusted. The full model was adjusted for age, sex, paternal pre-pregnancy BMI status, delivery mode, breastfeeding duration, age at AP, BMI at AP, and BMI at AR. *P*-value in bold font indicates a significant difference

### The association between positive health behaviours and the risks or likelihoods of having overweight or obesity in the first grade of primary school for children with a late AP or an early AR

As presented in Table 4, for children with a late AP, those with a high PA level were less likely to be overweight in the first grade (OR: 0.45, 95% CI: 0.20 to 0.99, *P* = 0.04). In the sensitivity analyses, for children with an early AR, limited screen time was linked with decreased risk of overweight or obesity (*RR*: 0.69, 95% CI: 0.52 to 0.93, *P* = 0.02). There was no evidence for associations between other positive health behaviours and overweight or obesity in children with a late AP or an early AR.

**Table 4:** Association between positive health behaviours and overweight or obesity in first grade of primary school for children with a late AP or an early AR

Health behaviour	Children with a late AP, OR (95% CI)	Children with an early AR, RR (95% CI)	
	**Overweight (*n* = 220)**	**Overweight (*n* = 297)**	**Overweight or obesity (*n* = 408)**
**Processed food**	reference		
**Traditional food**	1.07 (0.67–1.70)	1.21 (0.89–1.65)	1.11 (0.88–1.42)
**Light meal**	0.80 (0.32, 2.05)	1.10 (0.62–1.95)	0.91 (0.56–1.45)
**Low PA level**	reference		
**Moderate PA level**	0.82 (0.48–1.38)	1.29 (0.94–1.77)	1.29 (0.99–1.67)
**High PA level**	**0.45 (0.20–0.99)**	0.88 (0.53–1.48)	0.87 (0.59–1.28)
**High sleep quality**	1.19 (0.67–2.13)	1.02 (0.72–1.45)	1.10 (0.85–1.43)
**Sufficient sleep**	0.82 (0.50–1.35)	1.10 (0.78–1.54)	1.11 (0.85–1.43)
**Non-late-night snacking habit**	0.80 (0.30–2.08)	0.99 (0.72–1.37)	0.96 (0.76–1.23)
**Limited screen time**	1.45 (0.79–2.63)	0.74 (0.50–1.06)	**0.69 (0.52–0.93)**

*OR* odds ratio, *RR* risk ratio, *CI* confidence interval, *PA* physical activity. The adjusted covariates included: age, sex, birth weight, gestational age (preterm birth), delivery mode, breastfeeding duration, parental ages at birth, parental pre-pregnancy BMI statuses, parental education levels, perceived household income level, number of family numbers, number of siblings, dietary pattern, physical activity level, sleep disturbances, sleep duration, screen time, late-night snacking habit, and BMI magnitude at AP and AR. Value in bold font indicates a significant difference

A control analysis was also conducted to confirm whether these positive health behaviours were associated with a lower likelihood of developing overweight or obesity in children with a non-late AP or a non-early AR. The results in Table 5 only indicated that children with a non-early AR had a lower likelihood of developing overweight in the first grade if they did not have late-night snacking habits (OR: 0.04, 95% CI: 0.00 to 0.49, *P* = 0.01). The results of the sensitivity analyses also confirmed the association (OR: 0.06, 95% CI: 0.01 to 0.63, *P* = 0.02). Nevertheless, we did not observe the association in children with an early AR. Meanwhile, there was no evidence for associations between other positive health behaviours and overweight or obesity in children with a non-late AP or non-early AR.

**Table 5:** Association between positive health behaviours and overweight or obesity in first grade of primary school for children with a non-late AP or non-early AR

Health behaviour	Children with non-late AP, OR (95% CI)	Children with non-early AR, OR (95% CI)	
	**Overweight (*n* = 27)**	**Overweight (*n* = 13)**	**Overweight or obesity (*n* = 18)**
**Processed food**	reference		
**Traditional food**	0.24 (0.04–1.43)	1.82 (0.21–15.64)	2.50 (0.28–22.53)
**Light meal**	1.74 (0.12–26.16)	NA	
**Low PA level**	reference		
**Moderate PA level**	4.29 (0.89–20.73)	0.28 (0.02–4.08)	0.82 (0.09–7.51)
**High PA level**	1.48 (0.18–12.46)	0.11 (0.00–2.96)	0.46 (0.02–10.38)
**High sleep quality**	0.51 (0.08–3.45)	NA	NA
**Sufficient sleep**	0.48 (0.07–3.33)	0.41 (0.02–8.33)	1.59 (0.10–25.00)
**Non-late-night snacking habit**	0.37 (0.08–1.69)	**0.04 (0.00–0.49)**	**0.06 (0.01–0.63)**
**Limited screen time**	2.13 (0.15–33.33)	3.80 (0.14–10.00)	4.17 (0.36–10.00)

*OR* odds ratio, *CI* confidence interval, *PA* physical activity, *NA* not applicable. The adjusted covariates included: age, sex, birth weight, gestational age (preterm birth), delivery mode, breastfeeding duration, parental ages at birth, parental pre-pregnancy BMI statuses, parental education levels, perceived household income level, number of family numbers, number of siblings, dietary pattern, physical activity level, sleep disturbances, sleep duration, screen time, late-night snacking habit, and BMI magnitude at AP and AR. Value in bold font indicates a significant difference

### Discussion

This was a longitudinal study that involved more than 2000 children born after 2010 in Shanghai, China. We linked the estimated AP and AR timing to bodyweight outcomes in their first year of primary school and identified that a late AP or an early AR was associated with a higher risk of overweight but not obesity in this population. For children with a late AP, a high PA level was associated with a lower likelihood of developing overweight in the first grade, and for children with an early AR, limited screen time showed an association with a decreased risk of overweight or obesity in the first grade. On the other hand, although children with a non-early AR who did not have late-night snacking habits had a lower likelihood of developing overweight in the first grade, this association was not observed in children with an early AR.

The instant outcome of an early AR or the longitudinal association between AP timing and the risk of adiposity in school-age children has been scarcely reported [49–54]. A late AP may reflect a slow growth during infancy due to undernutrition, which may program a thrifty metabolism that exerts adverse effects later in life, especially if a growing child is exposed to overnutrition [42]. One of the adverse effects during middle childhood is overweight, which was observed in our study. Previous studies also confirmed that a late AP tended to result in an increased risk of overweight or obesity (at the age of six years) [43]. However, the data in Table 1 show that the detection rate of obesity in children with a late AP was far less than that in children with a non-late AP, which indicated a lower risk of obesity in the first grade in children with a late AP. Inconsistency is noticeable regarding the role of a late AP on overweight or obesity during childhood, and more studies are encouraged to clarify this association.

Children who underwent an early AR were found to gain fat faster than children who rebounded later [42, 80]. An early AR was associated with low fatness before the rebound and increased fatness development after the rebound, which is a consequence of reduced energy in early life followed by increased fat intake with age [42]. Several studies reported the health outcome of developing overweight later in life with an early occurrence of AR. They supported our result that an early AR was associated with the likelihood of having overweight at young school-age [80, 81]. Nevertheless, a French study found an association between an early AR and a higher likelihood of developing obesity at the same age [52]. This association was not observed in our population, whereas we noticed that the covariates introduced into the adjusted models were different, and we additionally introduced the BMI magnitude at the occurrence of AP and AR. The timing of AR was identified as a critical window for predicting childhood weight status, but the susceptibility of the predictor needs further exploration.

We also explored whether positive health behaviours were associated with a lower risk/odds of overweight or obesity during middle childhood among children at risk of adiposity (children with a late AP or an early AR) and observed that high-level PA might link to the lower odds of overweight in children with a late AP. PA plays a prominent role in the regulation of energy expenditure since it is the only activity that is totally under conscious control [82]. Moderate- or high-level PA is generally recognized as the answer to controlling overweight and obesity in children [83, 84]. Our study adds to the epidemiological evidence that high-level PA might even help to reduce the likelihood of overweight among children at risk of adiposity. Meanwhile, we observed that limited screen time might help reduce the risk of overweight or obesity among children with an early AR. The mechanisms include increased eating while using screens, viewing advertising for high-calorie, low-nutrition foods and beverages, and disrupting sleep [85]. Randomized controlled trials of limiting screen time have reported reduced weight gain in children, demonstrating a cause-and-effect relationship [85]. We provide additional evidence that limiting screen time could even have an effect on adiposity prevention among children at risk of overweight or obesity (children with an early AR). Moreover, late-night snacking habits were reported to be associated with poor sleep, skipping breakfast, higher calorie consumption at dinner, and increased BMI or obesity in children [86, 87]. In this study, for children with a non-early AR (children at low risk of adiposity), those who did not have late-night snacking habits had a reduced likelihood of having overweight or obesity. However, we did not observe this association in children with an early AR (children at high risk of adiposity). Thus, it suggested that although the positive habit of non-late-night snacking showed its potential effect in preventing overweight or obesity during middle childhood in low-risk children, it might be less effective in children with an early AR.

The general goals of positive health behaviours are to maintain a healthy BMI, and this is why we included behaviours that involved eating, PA, sleep, and screen time. Nevertheless, our results indicated that most of these behaviours had no association with a lower likelihood of developing adiposity in the first grade in children at risk of overweight or obesity (children with a late AP or an early AR). On the one hand, the non-significance in those expected associations may be related to the small sample size that was available for analysis. On the other hand, although our observational study design limits the causal inferences, a causal relationship may exist in which a late AP and an early AR are insurmountable stumbling obstacles for overweight and obesity prevention among children [88–90]. Combined with the previous data, we speculate that intervention approaches for adiposity in children with a late AP or an early AR are mainly ineffective. Hence, a plan B for prevention can move forward for the prevention of a late AP and an early AR, and an effective preventive approach is urgently needed. Interventions tailored to children with a late AP or an early AR at an early age can help prevent or limit excess weight gain before obesity becomes irreversible [26].

### Strengths and limitations

We had a relatively large number of subjects with data on growth and follow-up measurements. The period for our study was approximately seven years for each subject, and repeated anthropometric measurements were essential for the robustness of our results. Nevertheless, we note several limitations in our study. First, BMI trajectories in this study were estimated based on at least three measures per child during the first and 80th months of age. According to the condition, it is reasonable that all BMI measures of a child used to estimate the timing of AP and AR did not appear during infancy or near the school-age, which would exponentially increase the risk of bias to AP/AR timing estimation. Second, since the subjects were children who were born and brought up in an urban area in the most developed region, the generalizability of our findings to other populations with different socio-demographic backgrounds requires caution. Third, several factors, including underlying diseases, psychological conditions, and exposure to environmental chemicals, were not considered in our models. Fourth, the analyses on health behaviours were based on the hypothesis that these behaviours could be tracked from an early age onward. However, these behaviours may not be constant in the real world. The information in the FFQ, IPAQ, and CSHQ was parent-reported and based on recall over a period of one week or one month. Hence, measurement errors in these variables may have resulted in residual confounding to some extent. Furthermore, since an appropriate questionnaire for PA level measurements could not be identified for children in primary school, we adopted the IPAQ, even though its use is not recommended within the age group of our subjects. Additionally, the CSHQ is only a screening tool, so the sleep disturbances reported in our study were not diagnostic. Meanwhile, the choice of only including children with electronic questionnaires might skew the data. Fifth, since annual household income is a touchy subject for parents who fill out questionnaires, we only asked about perceived household income. However, perceived household income is not the same as actual household income. Sixth, we performed adjustments for some of the potential socio-demographic and behavioural confounders, but residual confounding still might have occurred, as in any observational study. Finally, this study tested associations but not causation. Therefore, our findings cannot be regarded as causal, and caution is needed in interpreting the results.

## Conclusion

In conclusion, our study reinforces the importance of the timings of AP and AR on the development of overweight in children in their first year of primary school. Since we did not observe the associations between all the selected positive health behaviours and a lower risk of having adiposity during middle childhood in our study, we suggested moving the prevention strategy to the early stage of life to control the occurrence of a late AP or an early AR.

## Supplementary Information

**Additional file 1: **

## Data Availability

The dataset used and/or analyzed during the current study is available from the corresponding author on reasonable request.
