# Supplementary material for: Longitudinal association between the timing of adiposity peak and rebound and overweight at seven years of age
Source: BMC Pediatr. 2022 Apr 19;22:215. doi: 10.1186/s12887-022-03190-9 (PMC9016949; doi:10.1186/s12887-022-03190-9)
Supplement: Supplementary file 1 — Additional file 1: [file 12887_2022_3190_MOESM1_ESM.docx]

| **Supplemental table 1 Total variance explained** | | | | | | | | | |
| --- | --- | --- | --- | --- | --- | --- | --- | --- | --- |
| Component | Initial eigenvalues | | | Extraction sums of squared loading | | | Rotation sums of squared loading | | |
|  | Total | % of variance | Cumulative % | Total | % of variance | Cumulative % | Total | % of variance | Cumulative % |
| 1 | 4.048 | 14.456 | 14.456 | 4.048 | 14.456 | 14.456 | 3.720 | 13.285 | 13.285 |
| 2 | 3.350 | 11.964 | 26.419 | 3.350 | 11.964 | 26.419 | 3.389 | 12.103 | 25.388 |
| 3 | 1.516 | 5.415 | 31.835 | 1.516 | 5.415 | 31.835 | 1.805 | 6.446 | 31.835 |

| **Supplemental table 2 Rotated component matrix** | | | |
| --- | --- | --- | --- |
|  | **Component** | | |
|  | **1** | **2** | **3** |
| Rice | 0.069 | **0.432** | -0.307 |
| Flour | 0.111 | **0.380** | 0.092 |
| Whole grains | -0.072 | **0.314** | **0.510** |
| Fried pasta | **0.505** | -0.071 | 0.258 |
| Fried meat | **0.649** | 0.012 | 0.089 |
| Fried vegetables | **0.596** | -0.051 | 0.117 |
| Smoked food | **0.386** | -0.018 | 0.065 |
| Pickled food | **0.309** | 0.048 | 0.052 |
| Poultry | 0.237 | **0.433** | 0.121 |
| Red meat | 0.103 | **0.558** | -0.117 |
| Fish and other fishery products | 0.133 | **0.530** | 0.183 |
| Egg | -0.073 | **0.580** | -0.033 |
| Fresh fruit | -0.099 | **0.504** | 0.202 |
| Fruit/vegetable juice | 0.124 | 0.118 | **0.691** |
| Fruit drinks | **0.364** | -0.028 | **0.579** |
| Carbonated drinks | **0.520** | -0.150 | 0.076 |
| Yoghurt | 0.076 | 0.233 | **0.342** |
| Milk and its products | 0.002 | **0.435** | -0.078 |
| Flavored milk drink | **0.407** | -0.037 | 0.285 |
| Fresh green leafy vegetables | -0.190 | **0.643** | 0.124 |
| Fresh yellow or red vegetables | -0.112 | **0.673** | 0.195 |
| Beans or soya products | 0.039 | **0.532** | 0.298 |
| Sugar or candy | **0.501** | 0.239 | -0.156 |
| Preserved fruit | **0.382** | 0.091 | 0.049 |
| Chocolate | **0.541** | 0.097 | -0.080 |
| Dessert | **0.451** | 0.252 | -0.160 |
| Puffed snacks | **0.675** | 0.018 | -0.087 |
| Fast food | **0.578** | -0.086 | 0.113 |

Three components were obtained: “processed” (high factor loadings, >0.3, high intakes of processed foods, such as fried foods, flavored drinks, and chocolate); “traditional” (high consumption of all types of red meat, poultry, vegetables, and fresh fruits); “light meal” (high loadings for grains, fruit juice, and yogurt).
